# Key Issues in the Development of an Evidence-Based Stratified Surgical Patient Safety Improvement Information System: Experience From a Multicenter Surgical Safety Program

**DOI:** 10.2196/13576

**Published:** 2019-06-24

**Authors:** Xiaochu Yu, Wei Han, Jingmei Jiang, Yipeng Wang, Shijie Xin, Shizheng Wu, Hong Sun, Zixing Wang, Yupei Zhao

**Affiliations:** 1 Peking Union Medical College Hospital, Chinese Academy of Medical Sciences Beijing China; 2 Department of Epidemiology and Biostatistics, Institute of Basic Medicine Sciences, Chinese Academy of Medical Science Beijing China; 3 The First Hospital of China Medical University Shenyang China; 4 Qinghai Provincial People’s Hospital Xining China; 5 Xiangya Hospital, Central South University Changsha China

**Keywords:** surgery, patient safety, information system, risk factors, evidence-based practice

## Abstract

Surgery is still far from being completely safe and reliable. Surgical safety has, therefore, been the focus of considerable attention over the last few decades, and there are a growing number of national drives to improve it. There are also a number of large surgical complication reporting systems and system-based interventions, both of which have made remarkable progress in the past two decades. These systems, however, have either mainly focused on reporting complications and played a limited role in guiding practice or have provided nonselective interventions to all patients, perhaps imposing unnecessary burdens on frontline medical staff. We have, therefore, developed an evidence-based stratified surgical safety information system based on a multicenter surgical safety improvement program. This study discusses some critical issues in the process of developing this information system, including (1) decisions about data gathering, (2) establishing and sharing knowledge, (3) developing functions for the system, (4) system implementation, and (5) evaluation and continuous improvement. Using examples drawn from the surgical safety improvement program, we have shown how this type of system can be fitted into day-to-day clinical practice and how it can guide medical practice by incorporating inherent patient-related risk and providing tailored interventions for patients with different levels of risk. We concluded that multidisciplinary collaboration, involving experts in health care (including senior staff in surgery, nursing, and anesthesia), data science, health care management, and health information technology, can help build an evidence-based stratified surgical patient safety improvement system. This can provide an information-intensified surgical safety learning platform and, therefore, benefit surgical patients by delivering tailored interventions and an integrated workflow.

## Introduction

Globally, each year, more than 230 million operations are performed and at least 7 million patients develop significant surgical complications, including 1 million perioperative deaths (at least half of which are preventable) [1,2]. Preventing harm to patients and improving the safety of surgical patients has, therefore, drawn considerable attention over the last few decades, and there are growing national drives to improve surgical patients’ safety [3]. There are also abundant opportunities for informatics-based improvements in perioperative care linked to the rapid development of information technology use in medical care. Information systems, such as computerized physician order entry, automated dispensing, barcode medication administration, electronic medication reconciliation, and personal health records, are playing an increasingly important role in enhancing patient safety by reducing medication errors [4,5]. Up to 50.2% (470/936) of medical errors can be avoided through the use of information systems [6]. However, most of these systems are not designed specifically for surgery. Their original intention was to regulate clinicians’ daily practice and allow health care providers to carry out routine jobs effectively, preventing potential errors [5]. Patient safety outcomes, which are crucial indicators for evaluating and improving surgical safety practice, cannot usually be obtained reliably through these systems. Extensive and carefully planned specialized information platforms for surgical sectors are, therefore, needed to collect data on patient safety [7]. Several nationwide surgical complication reporting and learning systems have, therefore, been developed in the past two decades, such as the United Kingdom’s National Reporting and Learning System [8]. This was established in late 2003 as a voluntary scheme for reporting patient safety incidents, to support learning from these incidents. Another large-scale Web-based information platform is the American College of Surgeons National Surgical Quality Improvement Program, which dates back to the 1980s and now incorporates hundreds of hospitals across the United States. It was developed to gauge the quality of surgical programs across different hospitals. The primary function of both of these systems is surgical incident reporting. Measuring incidence alone, however, is not enough to guide routine clinical safety behavior and enhance safety. A number of system-based interventions have, therefore, also been developed, mainly focusing on regulating clinical behavior and following the publication of *To Err is Human* [9]. These included the Surgical Patient Safety System (SURPASS) checklist, which requires 11 forms (nearly 100 items) to be completed and documented by providers for each individual undergoing surgery [10,11]. These intervention strategies regulate the daily clinical practices of health care staff and thus improve patient safety, but they impose a heavy workload in complex clinical settings. This could increase fatigue and undermine the adoption of and compliance with these systems by frontline health care staff [12]. The number of successful system implementations is, therefore, relatively small, with conflicting findings on their effect on patient safety. This tends to lead to skepticism about the true effectiveness of these systems and emphasizes the necessity of developing a patient safety system with high implementation efficiency and low operational complexity [13,14].

Surgical operations are complex procedures. The perioperative care process is a unique and challenging environment that requires close collaboration among surgeons, anesthetists, and nurses. Medical staff can also encounter sophisticated patient pathophysiological conditions. The length of patients’ stay in the hospital is relatively short in surgical departments, which has posed a significant challenge for surgeons in capturing the key factors influencing surgical outcomes and the timely transfer of key safety information to other members of the surgical team [15]. An information system capable of extracting knowledge from high volume and multi-sourced clinical data and supporting decisions in routine clinical operations could, therefore, improve efficiency and effectiveness. It could also provide a high degree of interoperability as well as information support, process management, and optimization in delivering evidence-based surgical safety interventions [16,17]. The complexities of the perioperative environment, however, can complicate the process of deployment and make technology implementation challenging. Some common issues in this environment must be addressed for successful deployment of information technology [18]. The development of this type of evidence-based patient safety information system (EPSIS), therefore, requires a holistic view. It needs to bring together clinical professionals, health care administrators, data scientists, and information technology engineers. This process can serve as an important catalyst in fostering a safety culture among frontline health workers, constructing a surgical safety ecosystem supported by data scientists, engineers, and administrators [19].

**Figure 1:**
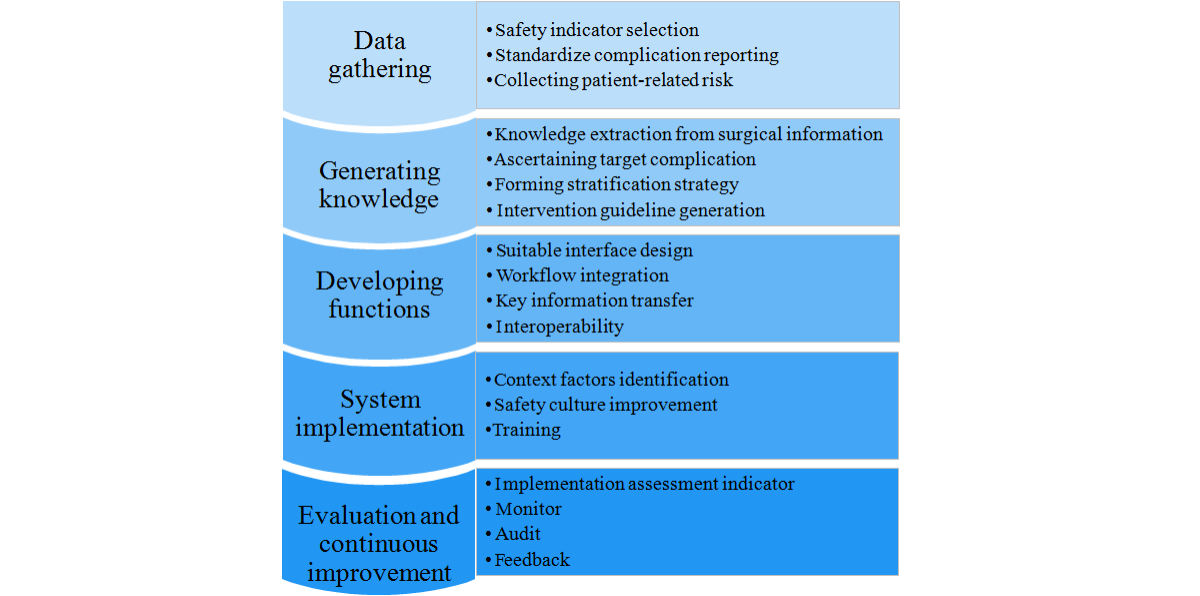
Critical issues in the life-cycle for designing and implementing an evidence-based surgical safety information system.

In 2014, a national project called Modern Surgery and Anesthesia Safety Management System Construction and Promotion (MSCP) was conducted in China. It aimed to improve perioperative patient safety. On the basis of this study, a perioperative surgical safety management information system was developed. This integrated patient data collection, processing, storage, and dissemination to support decision making, work control and documentation, and visualization [20]. To identify key elements and critical issues and formulate a framework to design, develop, and implement an EPSIS, a multidisciplinary panel of experts was assembled during the project period. This panel consisted of 10 medical experts, 5 nurses, 5 medical administrators, 3 data scientists, and 7 computer science engineers. All 30 experts attended several rounds of face-to-face consensus meetings, and widespread suggestions were collected from both the literature and panel members. Notes from project process meetings held by the central project group and the project executive groups from participating hospitals were also reviewed to identify practical challenges faced during the development of the project. These were refined and distributed to the panel members. Key methodological and implementation issues in designing and developing an evidence-based surgical safety information system were discussed, and recommendations on these issues were collected through these meetings. Finally, a summary of the framework was drafted and was circulated to panel members via email. Comments were collected until the group had reached a consensus. This paper discusses these issues (see Figure 1) in detail and uses practical examples from MSCP to illustrate them.

## Developing and Gathering Data

Clinical data are usually complex and highly distributed. This poses challenges in collecting data to support the development of an EPSIS, because this type of system relies heavily on the management of high-quality surgical safety data. Using high-quality data also supports workflow management, monitoring, and evaluation of the surgical patient safety system. To satisfy the needs of the whole information system, the accessibility, reliability, and timeliness of data should be ensured.

### Which Surgical Safety Indicators Should Be Collected?

Surgical safety promotion usually requires integrated interventions involving changes in a set of activities. These have long causal pathways and involve many factors that can influence the causal chain [21]. Recommended surgical safety indicators, therefore, include surgical complications and death and length of stays, which directly measure the observable harm. These indicators, unlike process or surrogate safety indicators (eg, error or culture), are identifiable and quantifiable and are more appropriate as natural endpoints in the *story* of patient safety [22]. The collection of data on these observable harms provides an easy way to investigate the causal chain. It could, therefore, help support learning about critical surgical safety issues linked to specific contexts as well as developing intuitive and target-sensitive functions for the safety improvement system [22-24].

### How Should Complication Reporting Be Standardized?

Information about complications is not always readily available. A complication reporting procedure requires accepted principles of accrual, display, and analysis of complication data to be predefined to capture complications in a structural way [22,25,26]. This will allow meaningful comparisons of the incidence of reported complications across different hospitals or different periods within the same hospital [27]. The subsequent data processing could also benefit from this structural reporting. Several classification criteria had been proposed, of which the Clavien-Dindo classification is the most widely used. However, this type of classification system provides limited reference in standardizing complication reporting because it mainly focuses on ranking complication categories in an objective and reproducible manner, on the basis of the therapy used to correct them [28]. In 2002, Martin et al proposed 10 criteria that should be met when reporting complications following surgery [29]. These proposals could serve as a reference in establishing the criteria for reporting information about complications.

### How Can We Retrieve and Integrate Information About Inherent Patient-Related Risk?

An incomplete data inventory leads to incomplete analyses. Electronic health records allow collection of particular elements of health-related information (eg, obesity, coronary heart disease, and hypertension) that are potentially associated with safety outcomes. However, it remains a challenge to form a multidisciplinary patient safety reference database because it needs to identify and track all data sources [30]. Knowledge of health data attributes, including data definitions, value sets, and other clinical coded content, is required. This means that the data retrieve process needs to involve information technology engineers, medical experts, and data scientists. Once the data have been captured, they can be filtered to support further clinical decision making by task and individual end user requirements. An additional step of data verification, introduced for data quality control, is also necessary. In MSCP, to ensure a trade-off between completeness and efficiency in information collection, a specialist panel was formed including medical experts, information engineers, and data scientists. By drawing on the literature and collecting expert opinions, this panel identified crucial information for inherent patient-related risk and determined how to obtain this information accurately and efficiently. To reduce user workload and transcription errors, a data extraction strategy was established by the clinical experts and information system engineers for data readily obtainable from hospital information systems. For information that is not routinely collected by hospital information systems, or which requires a special reporting mechanism because of its importance (such as complications), a stand-alone electronic data capture system was developed. This included 3 separate subsystems (ward, intensive care unit, and operation room). Patient information was entered by an established data entry team once the patients had been admitted. Complications (using clear definitions) were entered within a week of the patient’s discharge. The data collected from these 3 subsystems were centrally managed. Regular data quality audits were also conducted, and the results were reported monthly.

## Generating and Sharing Knowledge

Information alone is not enough to improve safety. Knowledge about the spectrum of complications and potential risk information extracted from the data is essential to formulate guidelines to help decision making about which patients to prioritize and what measures to take to prevent surgical complications. Ultimately, these data-driven decisions could play an auxiliary role in supporting clinical decisions and lead to more effective and appropriate use of resources through better procedures. New data obtained through the system are likely to stimulate the updating of knowledge and, therefore, further improve decisions.

### How Should We Prioritize Complications to Target?

Not all complications are equally important to patient safety. For instance, some complications have an extremely low rate of incidence (eg, pulmonary torsion), are not related to severe harm (eg, subcutaneous hematoma), or are not sensitive to prevention measures (eg, hypothyroidism after thyroidectomy). To ensure that the system is both operationally feasible and cost-effective in routine clinical practice, it is important to prioritize complications. We recommended prioritizing complications based on high incidence and serious prognosis and which are more likely to be preventable. We also suggested that it was important to consider local conditions. Table 1 shows an example from the MSCP Project, using empirical data and expert consensus to identify surgical complications with these 3 characteristics. Complications with low incidence can still be collected through the system. Increased experience and evidence may enable groups to identify underlining patterns for the occurrence of these complications and targeted intervention measures can then be formulated.

### How Can We Translate Surgical Safety Information Into an Evidence-Based Stratifying Strategy?

An evidence-based stratifying strategy implies that patients with different risk factors (identified using data collected and consensus among the expert panel) will receive hierarchical and targeted interventions. The ability to make a preoperative determination of the overall risk for multiple major complications is a prerequisite for clinical decision making and securing surgical patients’ safety, which is the ultimate goal of EPSIS [31-33]. Using the concept of *risk population* from epidemiology, we defined all surgical patients as the *risk population* for surgical complications at the stage before surgery [20]. In other words, every surgical patient could potentially develop any kind of complication and their risk of doing so is determined by factors that vary among the patients. Knowledge about patients’ existing risk factors can be generated using a series of computational models to translate input data [34]. This means that major contributory factors to complications can be identified and intervention measures can then be developed for patients.

Oinas-Kukkonen argued that intervention should be “tailored to the potential needs, interests, personality, usage context, or other factors relevant to a user group” and that a system that offers personalized content or services has greater effectiveness and efficiency [35]. Patients can be managed through the system on the basis of their identified inherent risk. Specific interventions can be offered to patients with different levels of risk. This can, therefore, determine the appropriate amount of resources for each surgical patient, which is vital for optimizing patient flow. Examples of this concept include the well-known Physiological and Operative Severity Score for the enUmeration of Mortality and Morbidity scoring system and those recently developed by the National Surgical Quality Improvement Program in the United States [25,36]. With the growing tendency for medical professionals to become more specialized, using the risk population concept in the surgical safety field can also provide a macroscopic view across safety issues in multiple specialties. It, therefore, provides a more global picture for system-wide intervention planning.

### How Can We Formulate Targeted Intervention Guidelines?

**Figure 2:**
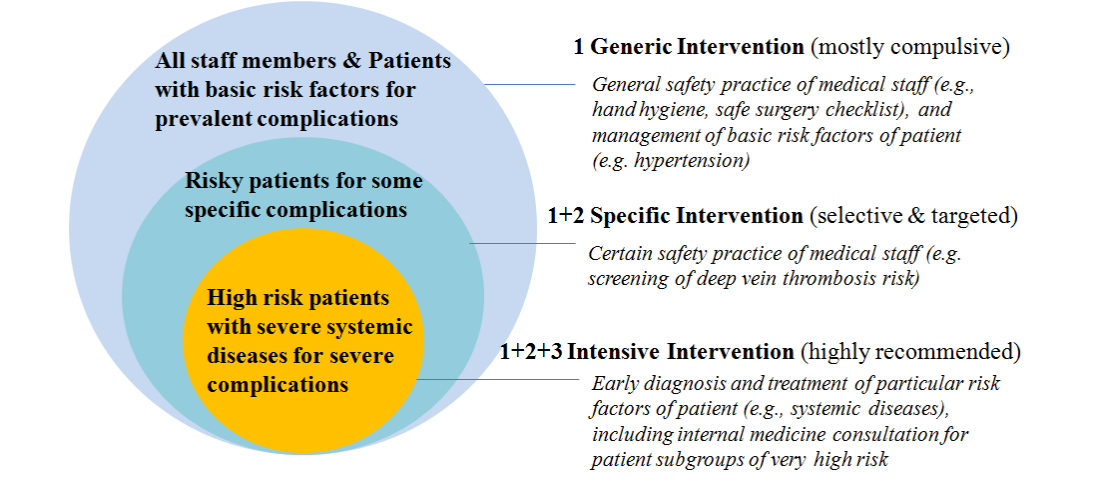
Stratified intervention for patients at different risk level of complications.

A system-integrated intervention guideline for decision making should map out which specific surgical safety intervention measures should be provided to patients with particular baseline risk and how the intervention should be carried out. The evidence from theory, the literature, and empirical research could be brought together and developed through standard procedures, such as the Delphi process [37]. Frontline staff and senior management, including both administrative and clinical leaders, should be involved in formulating the guidelines to get a buy-in from different professional groups (eg, surgeons, nurses, and anesthesiologists) to use the system to deliver interventions [14,38]. For instance, in MSCP, the interdisciplinary team of centralized researchers and clinicians reviewed the relevant research to identify interventions with the greatest benefit and the lowest barriers to use. Figure 2 shows how patients, complications, and interventions are provided in a stratified way in this system.

**Table 1:** Examples of prioritization of complications in the modern surgery and anesthesia safety management system construction and promotion project.

Complication	Decision
Surgical site infection	A^a^
Delayed healing or nonhealing incision	A
On ventilator ≥48 hours	A
Death or confirmed death	A
Hypoparathyroidism	B^b^
Coma after operation ≥24 hours	B
Respiratory failure	A
Electrolyte disturbance	B
Urinary tract infection	A
Pleural effusion	B
Acute renal failure	A
Skull defect	B
Cerebral edema	B
Hemorrhage requiring ≥4U RBC infusion 72 hours postoperatively	A
Iatrogenic pneumothorax	C^c^
Esophagus anastomotic fistula	C
Aphasia	C
Stress ulcer	C
Pneumocrania	C
Myasthenia crisis	C
Vocal cord paralysis	C
Pulmonary torsion	C
Secondary spinal canal stenosis	C
Fracture or loosening or dislocation of prosthesis	C
Internal or external fistula formation	C
Tracheal softening and collapse	C
Incisional hernia	C
Postoperative skin flap or subcutaneous effusion	C
Heterotopic ossification	C

^a^High incidence (≥0.5%), severe harm, and sensitive to prevention measure.

^b^High incidence, but not considered severe, or preventable based on the literature and an expert consensus.

^c^Very low incidence, based on complications reported through the system.

## Developing Functions and Applications

The data or applications should be presented in a visual form that users can understand. This makes them easy to use to support day-to-day operations and provide high-quality communication among hospital sectors for information- and knowledge-related functions [39]. Frontline medical staff and administrators at different levels should be extensively involved in the development process because adoption needs to be driven by clinicians where significant benefits can be articulated for them, the clinical and administrative teams, and the patients [40].

### How Can We Integrate the System Into the Existing Clinical Workflow and Make the System Sustainable?

The objective of EPSIS is improving surgical safety by optimizing rather than subverting the current workflow. However, the information system may be frustrating for frontline clinicians and organizations if it does not fit with existing systems. This is particularly true if it causes longer completion times and workflow disruptions [24,41]. The system should, therefore, be embedded into existing clinical routines in line with the way frontline medical staff like to work. It should also take into account the interdependencies among the health care staff, cultural environment, and the infrastructural organization of the hospital. The challenge in achieving this goal is to identify the critical elements in the surgical workflow that influence patient safety management and the need to exchange safety information among medical staff at a minimum cost to existing workflows and thus provide information support for the next task. System use by different people in the clinical setting may be improved by visually representing the workflow of a complex clinical work environment and using user-system interaction analysis and complex design changes. A picture will also help in imposing the necessary workflow control and has been shown to be more successful in changing an unsafe plan or preventing the omission of essential interventions [42,43]. For instance, *soft* or *hard* stop functions can be incorporated in the system. Soft stops can alert clinicians if the intervention is not carried out in line with the guidelines, and hard stops alert clinicians and stop the process unless the intervention has been completed or an explanation has been provided to the central control point to override the interception. Soft and hard stops are helpful in promoting a buy-in but can result in variation in practice and poor compliance with safety goals and intervention measures [44]. An example of soft stops from MSCP is an interception function developed to ensure the timely delivery of preoperative patient safety interventions. The surgery submission is not approved if the preoperative intervention has not been completed for the patient.

### How Can We Share Key Surgical Safety Information Smoothly and in a Timely Way?

Perioperative clinicians and staff have little opportunity to become familiar with surgical patients other than a quick determination of the required procedure. This lack of familiarity with and knowledge about patients could mean that perioperative team members (eg, operation room, postanesthesia care unit, and intensive care unit staff) might omit information that is important for surgical safety, such as allergies or antibiotic use. Integrated care and a high degree of interoperability should be highlighted to ensure high-quality communication of safety information among the various hospital sectors. This will allow quick and adequate responses about surgical patient safety issues [39]. Automatic reminders or alerts can facilitate the seamless transfer of vital information. They can also ensure that appropriate information is delivered to the surgeon at the right time and in a way that will ensure the surgeon receives and acts upon it. For instance, a reminder function was developed in MSCP to alert staff across the surgical ward and operation room when patients had high American Society of Anesthesiologists classifications.

### How Can We Make the User Interface Acceptable to End Users?

The interface is one of the most significant parts of an information system and helps users to work efficiently, effectively, and satisfactorily [40,45]. The interface design should aim to eliminate complexity, emphasize key elements, and use special colors to mark important areas. One tactic is to make the interface as similar as possible to the previous paper records, so that users do not need to search for the required fields. Only the field relevant to the current task should be displayed to the user, to increase efficiency. Feedback from end users should be sought and welcomed on an ongoing basis for the improvement of interface design and the iteration of development as a result.

### How Can We Achieve a High Level of Interoperability With Existing Information Systems and What is the Benefit?

Hospitals are information technology–intensive workplaces, incorporating many kinds of information systems (eg, electronic medical records, laboratory information management system, and office automation systems). These form an interoperable digital health ecosystem. Integration of the EPSIS with existing information systems could, therefore, influence several layers of caregivers and provide more convenient workflow control. Caregivers and professionals will be able to send, receive, find, and use digital health and care information in an appropriate, secure, timely, and reliable way and with little additional effort. Data interface standards should be established in the data-sharing process for patient-level data.

In MSCP, we integrated our intervention system into the existing hospital information system, which, we believe, provided considerable advantages in terms of the extent, depth, and value-added use of the intervention system [46]. For instance, being able to change intervention information electronically makes it easy to continuously monitor and validate the intervention behavior of medical staff. Integration is also helpful in avoiding the inconvenience of switching between different systems and, therefore, decreasing staff resistance to the intervention system [47]. Other benefits include the relative low learning cost, faster adoption, and ease of logging-in. However, there are also some barriers to integration with other information systems, such as the level of investment needed, additional data leakage risk, higher maintenance cost, and administrative resistance.

## System Implementation

There are two main challenges to managing the adoption, implementation, and sustainability of the system. The first is implementing the system organizationally, and the second is shaping the use of the system and related practices to achieve practical alignment with the intervention intention [48,49]. Drawing up an implementation plan and identifying and selecting appropriate methods or techniques that fit the context are considered fundamental to successful implementation [50].

### What Contextual Factors Influence System Implementation?

Ideas, practices, organizational arrangements, roles, and status in the information system all reflect and are influenced by the wider sociocultural context in which they occur [51]. This is particularly true for the organizational setting within which an information system is implemented, because it forms an integral part of that system. Several frameworks are available to identify contextual factors that are likely to influence the implementation of a given intervention [52,53]. Their use allows attention to be directed toward the contextual factors that are likely to hinder implementation as well as identifying facilitators of success [54,55]. For instance, Meijden et al identified 6 dimensions affecting implementation: (1) system quality, (2) information quality, (3) usage, (4) user satisfaction, (5) individual impact, and (6) organizational impact [56].

### How Should the Surgical Safety Culture and its Improvement Be Assessed?

Information system initiatives often fail because of mismatching between culture and the information system or a failure to understand culture and its influence on end user adoption of information systems [51]. Changing staff attitudes and views can help the staff understand the reasons for system changes and improve acceptance [57-59]. The use of tools, such as the Healthcare Research and Quality Hospital Survey on Patient Safety Culture, is recommended to inform perceptions about safety and related behavior, as well as to support the adoption of a safety intervention system [60-62]. However, culture change in a health care institution can be a long and intensive process requiring more cross-collaboration and greater user participation at all levels. Studies have suggested that organizational champions who can *shepherd* system implementation, influence cultural change, and act as a bridge with developers would be a valuable resource. Strategies such as printed educational materials, educational meetings, and educational outreach are also effective in changing attitudes and behaviors and increasing the use of a new information system [38,63].

### Training Strategy

The implementation of a new information system in a clinical setting often ignores the influence of processes and routines of clinical practice. A lack of understanding of system capabilities can lead to workarounds, with the new system being used in unintended ways [64]. A training plan must be designed and completed before the initial implementation and should include intensive support during implementation. Moreover, providing training shows the organization’s support in system implementation and development [65]. It is often effective to deliver training tailored to different users, for example, nurses, clinicians, and medical administrators. This should, however, include a holistic view of the entire system to strengthen the understanding of its function and goal. Hands-on practice and simulations may provide more benefit than only giving lectures [66]. It is also better to provide both compulsory and voluntary training elements [67]. Finally, training sessions are essential during or before system implementation, but ongoing training and development are also important [68]. Before the start of the MSCP project, training teams involving both clinicians and information technology engineers were established in each hospital. Unit-based education and training sessions were provided for nurses and other clinicians separately, because of the differences in workflow and the system operating interface. The standard operating procedure for efficient operation and compliance was translated into a course to facilitate the training process. Continuous training was also a part of MSCP, to reflect system updates and deployment of new function modules.

## Evaluation and Continuous Improvement

Surgical safety improvement is an iterative procedure. Postimplementation evaluation, feedback, and performance gap assessment can deepen insight into how and why particular changes did or did not occur. This can further increase the benefits of using the system, prolong its sustainability, and support dynamic learning and improvement [69,70].

### What Implementation Outcomes Should Be Used to Assess System Implementation?

When developing monitoring and evaluation plans for an EPSIS, new system implementation indicators (eg, usability) are recommended in addition to clinical outcomes (eg, complications, death, and length of stay). Different dimensions of outcomes and/or assessment methodologies have been proposed for evaluating the implementation of health care information systems [56,71-73]. For instance, Proctor et al distinguished among 3 distinct but interrelated types of outcomes for assessment in implementation studies: service (eg, safety), implementation (eg, fidelity), and client outcomes (eg, satisfaction) [73]. Hull et al outlined 8 implementation outcomes, defined as “the effects of deliberate and purposive actions to implement new treatments, practices, and services”. The outcomes were acceptability, adoption, appropriateness, feasibility, fidelity, implementation cost, penetration diffusion, and sustainability [74]. Correlating these implementation outcomes with implementation success and failure could strengthen the understanding of the mechanisms of the effect. This might further translate into evidence-based interventions to improve surgical safety.

### Monitoring, Auditing, and Formulating a Feedback Channel for Performance Improvement

A surgical safety information system needs monitoring and continuous auditing to ensure that it adheres to intervention guidelines [75]. Messaging functions could be used to enable real-time recording and transmission of any problems. In MSCP, the completion status of the surgical safety checklist for the operation room is recorded at different stages (sign in, time out, and sign out), and any violation at any stage is recorded and submitted.

Establishing a mechanism to provide feedback on the results of intervention protocol variations (eg, compliance and completeness) and patient outcomes to frontline medical staff and managers is essential for fostering a culture of surgical patient safety in hospitals [76,77]. These measures can encourage improvements in identifying and sharing information about patient safety incidents. They can also help the staff to identify problems during the implementation process, ultimately supporting continuous learning as well as increasing engagement of medical staff. For instance, case-enhanced learning can use real cases to allow staff to identify problems and solutions. This, therefore, provides learning resources about complications and clinical behaviors to support safety improvement. A redesign-action-feedback closed cycle can be formed among knowledge generation, function and application development, system implementation, and evaluation of effectiveness. This has no defined beginning and end point, and the cycle should not be interpreted as starting with prevention and ending with action.

In the MSCP project, a *graded confirmed awareness* subsystem was created. This gradually summarized patient outcomes and the implementation status of the system to improve awareness and confirmation in a specific sequence. This moved from patient to doctor and nurse in charge, to surgeon (daily), to department head (weekly), to medical affairs department (monthly), to hospital dean (quarterly), and then to permanent database. This system established dual duties (for subordinates and superiors) and dual supervision mechanisms (individual and external) for each person. Frontline staff and managers become equal elements in the chain securing patient safety. More importantly, this type of feedback channel could also help create a culture of patient safety and increase the motivation of hospital staff to deliver interventions through the system. In the MSCP, higher implementation rates were observed for all the postoperative prevention packages following the introduction of this subsystem (Table 2).

**Table 2:** Effects of implementing a Graded Confirmed Awareness System on surgeons in 4 hospitals in the modern surgery and anesthesia safety management system construction and promotion project.

Prevention category		Number with risk factors	Preoperative prevention measures delivered	Postoperative prevention measures delivered
**Before introduction of the graded confirmed awareness system, n (%)**			
	Generic intervention	11,472 (27.0)	10,671 (93.0)	5830 (50.8)
	Specific intervention	11,641 (31.7)	11,453 (98.4)	6905 (59.3)
	Intensive intervention	4588 (23.8)	3245 (70.7)	2064 (44.5)
**After introduction of the graded confirmed awareness system, n (%)**			
	Generic intervention	8947 (23.6)	7528 (84.1)	5535 (61.9)
	Specific intervention	8532 (23.2)	8270 (96.9)	6037 (70.8)
	Intensive intervention	3514 (20.1)	2241 (63.8)	1709 (48.6)

## Conclusions

**Figure 3:**
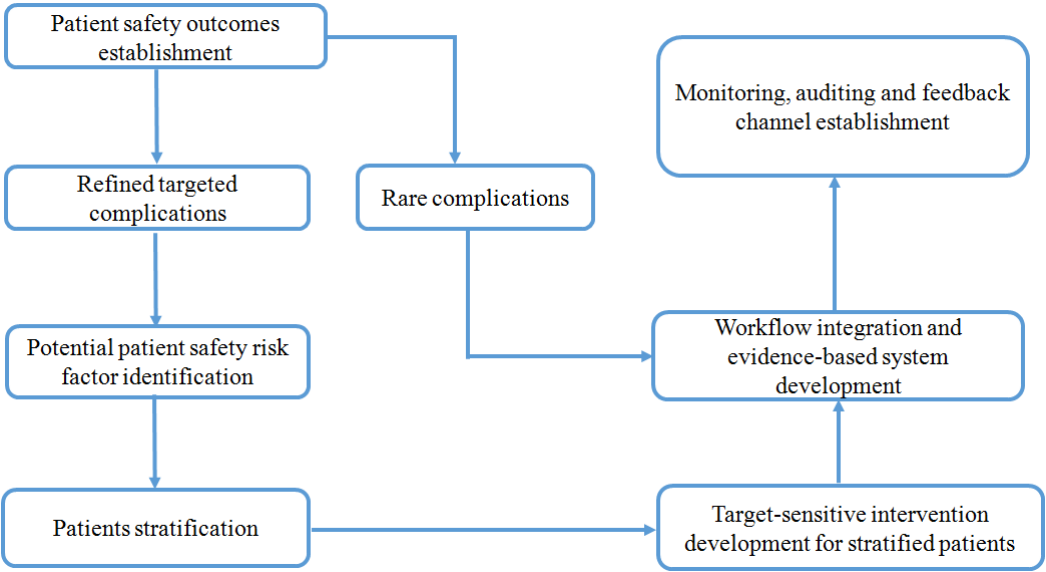
Design pattern rationale graph of an evidence-based surgical safety information system.

This study has outlined and discussed several critical issues in the process of developing an EPSIS. The design rationale of the system is summarized in Figure 3. The EPSIS is characterized by several distinct features, including the following:

1. Optimizing patient flow management and avoiding overload of medical staff by delivering tailored interventions to patients in a target-sensitive and stratified way based on inherent risk.
2. Incorporating the intervention into routine clinical activities and formulating a surgical safety information circle that ensures that critical safety and management information transfers smoothly between sectors and medical staff, in particular, senior managers, who play a pivotal role in this information circle.
3. Integrating the intervention information system with the existing hospital information system to construct an interoperable surgical safety improvement ecosystem, incorporating outcome reporting, intervention, monitoring, and feedback and finally developing a surgical safety learning system encouraging a patient safety culture in the hospital.

The issues we have discussed in this paper might serve as a reference for projects aiming to build an interactive mechanism to combine safety systems supporting wide and continuous improvement in surgical safety.

There are a number of limitations and challenges in constructing the EPSIS. The EPSIS in MSCP was mainly for patients admitted for elective surgery. Future expansion, for example, to emergency surgery and intensive care units, will present other challenges that have not been considered in this paper. It is difficult to establish evidence-based interventions for complications with low incidence in EPSIS, which is focused on complications. However, EPSIS is both a management system and learning platform. As more information is gathered, this problem will gradually be eliminated.

## Abbreviations

EPSIS: evidence-based patient safety information system
MSCP: modern surgery and anesthesia safety management system construction and promotion
